# Effect of oral magnesium supplement on cardiometabolic markers in people with prediabetes: a double blind randomized controlled clinical trial

**DOI:** 10.1038/s41598-022-20277-6

**Published:** 2022-10-28

**Authors:** Rezvan Salehidoost, Golshan Taghipour Boroujeni, Awat Feizi, Ashraf Aminorroaya, Masoud Amini

**Affiliations:** 1 Isfahan Endocrine and Metabolism Research Center, Isfahan University of Medical Sciences, Isfahan, Iran; 2 Internal Medicine Department, Medical University of Shahrekord, Shahrekord, Iran; 3 Biostatistics and Epidemiology Department, School of Public Health, Isfahan University of Medical Sciences, Isfahan, Iran; 4 Isfahan Endocrine and Metabolism Research Center, Isfahan University of Medical Sciences, Isfahan, 8187698191 Iran

**Keywords:** Diseases, Endocrinology, Medical research

## Abstract

To evaluate the effect of magnesium supplementation on insulin resistance and cardiovascular markers in people with prediabetes. A 12 week double-blind placebo-controlled randomized clinical trial was conducted at Isfahan Endocrine and Metabolism Research Center, Iran, on people with prediabetes (n = 86) to compare the effects of magnesium oxide 250 mg/day versus a placebo on anthropometric indices, blood pressure, fasting glucose, insulin, HOMA-IR index, C-reactive protein, uric acid and lipid profile. Both groups had similar distributions of anthropometric and biochemical variables at baseline. Those who received magnesium supplementation had significantly higher levels of HDL-cholesterol compared to the placebo group at the end of the study (49.7 ± 10.9 vs 43.6 ± 7.2 mg/dL, P = 0.003). The mean changes of HOMA-IR index, total cholesterol, LDL-cholesterol, triglyceride, uric acid and C-reactive protein levels as well as anthropometric indices and blood pressure in supplemented and placebo groups did not differ significantly. Magnesium supplementation increased HDL-cholesterol levels in people with prediabetes. However, other cardiometabolic markers were not improved by magnesium supplementation at the above dosage and duration.

## Introduction

Nowadays, lifestyle and human behavior have profoundly changed. People today are living longer, eating too much, and being inactive, leading to an increase in obesity and type 2 diabetes (T2DM) rates^1,2^. Thus, T2DM is listed as a global health and economic threat in the whole world^1^. Diabetes is the leading cause of kidney failure, blindness, and nontraumatic leg amputations as well as a leading cause of strokes and heart attacks^3–5^.

A key underlying mechanism of T2DM is the development of insulin resistance, and it is well known that normal glucose tolerance deteriorates gradually over time as beta cell function fails to compensate for the increased insulin resistance^6^. Prediabetes (impaired glucose tolerance [IGT] and/or impaired fasting glucose [IFG]) is the first stage of T2DM, in which blood sugar levels are higher than normal, but not yet at diabetes levels^2^. If prediabetes is detected early and treated effectively, the natural history of diabetes can be changed and complications can be delayed or prevented^2^.

There is evidence that mineral metabolism in individuals with glucose metabolic disorders is altered^7^. Hypomagnesemia is a common electrolyte disorder in patients with diabetes^8^. Magnesium plays a major role in many different biochemical reactions and helps to maintain a variety of vital functions, including muscle contraction, neuromuscular conduction, glucose regulation, myocardial electrical activity, and blood pressure^9,10^. Additionally, magnesium appears to play a substantial role in insulin secretion from pancreatic beta cells^11^. Therefore, some studies have investigated the effect of oral magnesium supplementation on glucose metabolism, insulin sensitivity and cardiometabolic markers in people at risk for developing diabetes^12–14^. The term cardiometabolic risk factors refers to circumstances that increase the risk of vascular events or diabetes^15^. In addition to traditional risk factors such as hypertension and dyslipidemia, this concept also includes emerging factors such as abdominal obesity, insulin resistance and inflammation.

There are very few well-designed intervention trials investigating the role of magnesium supplementation in the improvement of metabolic profile and insulin sensitivity in the people with prediabetes as a high risk group to develop diabetes. Moreover, the results are inconsistent, some showed that magnesium supplementation may improve metabolic profile and insulin sensitivity, whereas other studies did not confirm this^12–14^. In addition, According to our knowledge, there is no published data about the effect of magnesium supplementation on a wide range of the cardiometabolic markers in people with prediabetes simultaneously. Considering the literature at hand, we conducted a randomized clinical trial in people with prediabetes who are first-degree relatives of patients with T2DM as a high-risk group to evaluate the effect of oral magnesium supplementation on cardiometabolic markers.

## Materials and methods

This is a double-blind placebo-controlled randomized clinical trial conducted in Isfahan, Iran, from December 2018 through April 2019 at Isfahan Endocrine and Metabolism Research Center. The study was approved by Isfahan University of Medical Sciences ethics committee as IR.MUI.MED.REC.1397.252 and was registered at Iranian registry of clinical trials as IRCT20181226042129N1. Study participants gave informed consent before taking part in examinations and registrations, and the study was compliant with the Helsinki Declaration.

The participants were selected from an ongoing cohort study in Isfahan called the Isfahan Diabetes Prevention Study (IDPS) which was initiated in 2003^16^. The IDPS was performed on first-degree relatives of patients with T2DM to determine the potential risk factors for diabetes in people with a family history of T2DM as the primary risk factor for diabetes. Considering type I (α) and type II errors (β) 0.05 and 0.20 (statistical power = 80%), respectively, for detecting a mean difference (d) 1.4 and standard deviation 2.7 for HOMA-IR index as main study outcome^14^ between two groups; the sample size was estimated to be 68 patients. For covering the possible attrition during the study period, 86 patients (43 patients in each group) were included.

The inclusion criteria were as following: prediabetes status (5.6% ≤ HbA1c ≤ 6.4% or 100 mg/dL ≤ FPG < 126 mg/dL)^17^, between ages 18 to 65, glomerular filtration rate greater than 60 mL/min, blood pressure less than 140/90 mmHg, and willingness to maintain current diet and physical activities patterns during the intervention period. Exclusion criteria included taking magnesium supplements in the past 6 months, taking anti-diabetes medications, antihypertensives or diuretics, smoking, willing to take any kind of supplements during the study except those provided by the study, smoking, being pregnant, having a history of cardiovascular disease, renal disease, cancer, inflammatory bowel disease, pancreatitis, chronic diarrhea or any type of the chronic diseases, or inability to provide written informed consent. Magnesium supplements are generally considered safe for most people when taken appropriately. In some people, magnesium might cause digestive issues such as diarrhea, nausea, and flatulence. Under certain circumstances, such as renal disease, taking diuretics and antihypertensives, and chronic diarrhea, magnesium may be more unsafe. Therefore, all these conditions were considered as exclusion criteria.

The 86 subjects who were selected to participate in the study were randomly allocated to receive either 250 mg magnesium oxide tablet or placebo containing non magnesium ingredients once daily for 12 weeks. Random allocation was done using permuted block randomization with block size 4 through random digits generated by using SPSS software from a uniform distribution (0–1).

Magnesium oxide tablets containing 250 mg magnesium and the dummy starch-based placebo tablets were prepared and labeled by Jalinus Pharmaceutical Company in Tehran, Iran, according to a randomization scheme. Both patients and investigators were blinded about the used treatments by providing the magnesium supplement and placebo in tablets matched in shape, size, taste, odor and color. For random concealment, the randomization codes were held by independent researcher who was not involved in the study. The measurements were done by persons who were not aware of the randomization of the participants.

The participants were invited to the clinic in the morning after overnight fasting. Height, weight, waist, and arm circumference were measured using standard techniques^18^, with the patients in light clothes and without shoes. Waist circumference was measured midway between the lower rib margin and the iliac crest at the end of a gentle expiration. Arm circumference was measured at the middle tip of the shoulder and elbow. Body mass index (BMI) was calculated by dividing weight as kg to square of height as meter. Resting blood pressure was measured after the subjects had been seated for 10 min, using standard techniques^18^. Subsequently, 10 cc venous blood was sampled from anterior cubital vein after 12 h fasting. Blood samples were evaluated at Isfahan Endocrine and Metabolism Research Center laboratory. The primary outcomes were changes in blood pressure, lipid profile and glucose levels from baseline to 12 weeks. Additionally, the secondary outcomes included changes in anthropometric indices, HOMA-IR index, C-reactive protein and uric acid.

During the study, participants were advised not to alter their usual physical activities or dietary habits. In addition, they were asked not to use any supplements except those provided to them in the research. During the intervention, the adherence to the study protocol was checked monthly. Participants are warned about the study protocol if they do not consume their medication or change their lifestyle completely. The unused medicine was counted at the end of each month of the study, and if 10% of tablets (3 days in a month) were not used, the patient was excluded. After 12 weeks of treatment, anthropometric variables and blood tests were measured again.

Fasting plasma insulin concentration was assayed by chemiluminescence. Inter and intra assay coefficient of variations (CVS) for plasma insulin was 5.8% and 2.9%, respectively. Fasting plasma glucose (FPG), triglyceride (TG), total cholesterol, low density lipoprotein (LDL)—cholesterol, high density lipoprotein (HDL)—cholesterol and uric acid were measured by direct enzymatic colorimetric. Inter and intra assay CVS for fasting plasma glucose and lipid profile measurement were less than 5%. Inter and intra assay CVS for uric acid were 5% and 2%, respectively. HbA1c was measured by liquid chromatography. C-reactive protein (CRP) was measured by Immunoturbidimetry. Inter and intra assay CVS for CRP were 4.4% and 2.8%, respectively. HOMA-IR index was calculated based on the following formula as fasting plasma glucose (mg/dL) multiplied to fasting plasma insulin (μU/L) divided by 405^19^. Pars Azmoon kit made in IRAN were used for all blood tests.

### Statistical analysis

Continuous and categorical data were presented as mean (SD) and frequency (percentage), respectively. Normality of continuous data was evaluated using Kolmogorov–Smirnov test and Q-Q plot. The baseline continuous and categorical data were compared using independent samples t-test and Chi-squared test, respectively. The mean values of main outcomes before and after intervention was compared using paired samples t-test. Adjustment was made for baseline values of main outcome and Analysis of Covariance (ANCOVA) was used for comparing the mean values after intervention. All statistical analyses were done using SPSS version 16 (SPSS, Inc., Chicago IL, USA), and P < 0.05, was considered as statistically significant.

### Ethical approval

All procedures performed in studies involving human participants were in accordance with the ethical standards of the institutional and/or national research committee and with the 1964 Helsinki declaration and its later amendments or comparable ethical standards. The study was approved by the ethics Committee of the Isfahan University of Medical Sciences.

### Informed consent

Informed consent was obtained from all patients for precipitation and registration.

## Results

**Figure 1:**
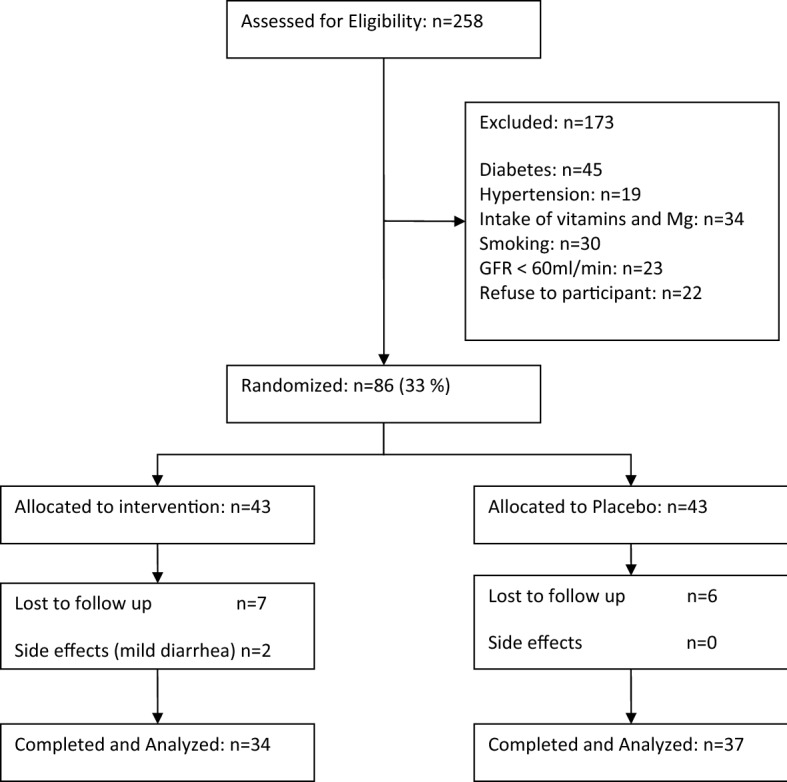
Flow diagram of participants.

Among those patients who assigned to magnesium supplement or placebo groups, 13 patients lost their follow-up (6 patients in placebo and 7 patients in magnesium-supplemented group) and 2 patients in the magnesium-supplemented group complained of mild diarrhea as a side effects of magnesium consumption and stopped the drug, so were excluded. Finally, 71 people completed the study, 34 in the magnesium-supplemented group and 37 in the placebo group (Fig. 1).

The baseline characteristics of two groups are shown in Table 1. The mean age of the total participants (78.9% female and 21.1% male) was 55.7 year. The mean age of the magnesium-supplemented group (82.4% female and 17.6% male) and placebo (75.7% female and 24.3% male) groups were 56.7 and 54.8, respectively. The distribution of age (P = 0.14) and sex (P = 0.57) were comparable between magnesium-supplemented and placebo groups. Other parameters were also similarly distributed between two groups (All P > 0.05).

**Table 1:** Baseline characteristics of the study participants.

Variables	Placebo group (n = 37)	Magnesium-supplemented group (n = 34)	P value*
Age (year)	54.8 ± 4.9	56.7 ± 5.9	0.14
Sex (female/male)	F: 28; M:9	F: 28; M:6	0.57
Weight (kg)	79.6 ± 15.9	75.2 ± 7.3	0.12
Waist circumference (cm)	100.6 ± 6.3	100.5 ± 11.3	0.96
Arm circumference (cm)	32.1 ± 3.54	32.3 ± 2.9	0.95
BMI (kg/m^2^)	30.7 ± 5.1	30.0 ± 3.3	0.51
SBP (mmHg)	120.5 ± 13.3	120.0 ± 20.1	0.85
DBP (mmHg)	79.2 ± 15.7	76.8 ± 13.1	0.62
FPG (mg/dL)	112.5 ± 12.2	115.1 ± 14.2	0.57
Fasting plasma insulin (μU/mL)	14.8 ± 9.6	8.2 ± 5.9	0.08
HBA1c (%)	5.8 ± 0.6	5.8 ± 0.7	0.63
HOMA-IR index	4.0 ± 2.8	3.1 ± 4.4	0.17
Triglyceride (mg/dL)	158.0 ± 65.3	156.4 ± 77.9	0.58
Total cholesterol (mg/dL)	184.0 ± 41.0	193.1 ± 37.5	0.32
HDL-cholesterol (mg/dL)	42.9 ± 7.8	46.0 ± 11.8	0.11
LDL-cholesterol (mg/dL)	97.7 ± 27.4	99.9 ± 27.3	0.54
Uric acid (mg/dL)	4.8 ± 1.1	5.2 ± 1.4	0.20
CRP (mg/L)	1.3 ± 2.6	1.3 ± 2.7	0.91

*BMI* body mass index, *SBP* systolic blood pressure, *DBP* diastolic blood pressure, *FPG* fasting plasma glucose, *CRP* C-reactive protein.

*Resulted from independent samples t-test for continuous and chi-squared for categorical variables.

Anthropometric characteristics of participants before and after intervention are presented in Table 2. A significant decrease in weight and BMI was observed in the both magnesium-supplemented and placebo groups at the end of study. However, the mean changes between the two groups did not differ significantly. Additionally, the waist and arm circumference did not alter significantly with intervention in both groups.

**Table 2:** Anthropometric indices of participants before and after intervention.

	Magnesium-supplemented group				Placebo group				Difference
	Baseline	End	Mean Difference ± SE	P value*	Baseline	End	Mean Difference ± SE	P value*	P value**
Weight (kg)	75.2 ± 7.3	74.2 ± 7.2	− 0.9 ± 0.4	0.04	79.6 ± 15.9	78.2 ± 16.5	− 1.6 ± 0.6	< 0.001	0.42
BMI (kg/m^2^)	30.0 ± 3.3	29.6 ± 3.3	− 0.4 ± 0.2	0.03	30.7 ± 5.1	30.1 ± 5.4	− 0.6 ± 0.2	< 0.001	0.41
WC (cm)	100.5 ± 11.3	99.7 ± 11.8	− 0.9 ± 2.8	0.06	100.6 ± 6.3	100.3 ± 6.7	− 0.3 ± 1.9	0.44	0.35
AC (cm)	32.3 ± 2.9	32.7 ± 6.5	0.2 ± 1.1	0.84	32.1 ± 3.5	32.0 ± 4.5	− 0.2 ± 0.5	0.75	0.73

*BMI* body mass index, *WC* waist circumference, *AC* arm circumference.

*Resulted from paired t-test for comparing mean values of outcomes before and after intervention in each study group.

**Resulted from ANCOVA for comparing two study groups based on mean values of outcomes after intervention and adjustment for baseline values.

Cardiometabolic markers of participants are shown in Table 3 before and after intervention. Those who received magnesium supplementation had significantly higher levels of HDL-cholesterol compared to the placebo group at the end of the study (49.7 ± 10.9 vs 43.6 ± 7.2 mg/dL, P = 0.003; estimated statistical power 0.90). Triglyceride levels improved in the both magnesium-supplemented and placebo groups (156.4 ± 77.9 mg/dL to 123.1 ± 48.4 mg/dL, P = 0.03 and 158.0 ± 65.3 mg/dL to 135.7 ± 51.7 mg/dL, P = 0.02). However, the mean change between the two groups did not differ significantly (P = 0.283). After 12 weeks, fasting insulin, HbA1c and CRP levels, and HOMA-IR index all significantly increased in both groups, but mean changes between the two groups were not significantly different. Systolic blood pressure, diastolic blood pressure, total cholesterol, LDL-cholesterol and uric acid did not change significantly in magnesium-supplemented nor in placebo groups.

**Table 3:** Cardiometabolic markers of participants before and after intervention.

	Magnesium-supplemented group				Placebo group				Difference
	Before	After	Mean difference ± SE	P value*	Before	After	Mean difference ± SE	P value*	P value**
SBP (mmHg)	120.0 ± 20.1	115.7 ± 12.8	− 4.2 ± 4.03	0.30	120.5 ± 13.3	113 ± 24.5	− 7.5 ± 3.7	0.05	0.55
DBP (mmHg)	76.8 ± 13.1	73.9 ± 9.2	− 2. ± 3.2	0.38	79.2 ± 15.7	74.1 ± 13.0	− 5.1 ± 3.2	0.12	0.98
Total-C (mg/dL)	193.1 ± 37.5	188.2 ± 38.7	− 4.9 ± 8.7	0.58	183.6 ± 40.8	176.9 ± 35.5	− 6.7 ± 6.6	0.32	0.36
TG (mg/dL)	156.4 ± 77.9	123.1 ± 48.4	− 33.3 ± 14.0	0.02	158.0 ± 65.3	135.7 ± 51.7	− 22.3 ± 8.9	0.01	0.28
HDL-C (mg/dL)	46.0 ± 11.8	49.7 ± 10.9	3.7 ± 1.1	0.002	42.9 ± 7.8	43.6 ± 7.2	0.7 ± 0.7	0.29	0.003
LDL-C (mg/dL)	99.9 ± 27.3	95.0 ± 24.1	− 4.9 ± 5.3	0.37	97.7 ± 27.4	88.2 ± 22.2	− 9.5 ± 4.2	0.03	0.25
HbA1c (%)	5.8 ± 0.7	6.6 ± 0.8	0.8 ± 0.1	< 0.001	5.8 ± 0.6	6.5 ± 0.8	0.7 ± 0.1	< 0.001	0.55
FPG (mg/dL)	115.1 ± 14.2	116.5 ± 13.7	− 1.5 ± 2.0	0.48	112.5 ± 12.2	111.8 ± 14.3	− 0.7 ± 1.8	0.72	0.29
FPI (μU/mL)	8.2 ± 5.9	16.9 ± 7.8	8.7 ± 1.4	< 0.001	14.8 ± 9.6	17.9 ± 8.4	3.2 ± 1.3	0.02	0.18
Uric acid (mg/dL)	5.2 ± 1.4	4.9 ± 1.1	− 0.2 ± 0.2	0.28	4.8 ± 1.1	5.1 ± 1.0	0.26 ± 0.1	0.04	0.09
CRP (mg/L)	1.3 ± 2.7	3.1 ± 4.0	1.8 ± 0.5	0.001	1.3 ± 2.6	2.2 ± 2.1	0.8 ± 0.4	0.03	0.10
HOMA-IR index	3.1 ± 4.4	4.9 ± 2.5	1.8 ± 0.9	0.06	4.0 ± 2.8	4.9 ± 2.6	0.9 ± 0.4	0.02	0.80

*SBP* systolic blood pressure, *DBP* diastolic blood pressure, *C* cholesterol, *TG* triglyceride, *FPG* fasting plasma glucose, fasting plasma insulin, *CRP* C-reactive protein.

*Resulted from paired t-test for comparing mean values of outcomes before and after intervention in each study group.

**Resulted from ANCOVA for comparing two study groups based on mean values of outcomes after intervention and adjustment for baseline values.

## Discussion

In this randomized controlled trial (RCT) involving people with prediabetes, we found that magnesium supplementation 250 mg daily for 12 weeks increased HDL-cholesterol, but no effects on total cholesterol, LDL-cholesterol and triglyceride concentrations. Also, no effect was observed on fasting plasma glucose and insulin levels, HbA1c and HOMA-IR index as well as uric acid and CRP levels following magnesium supplementation.

The positive effect on HDL-cholesterol is consistent with several, but not all, RCTs investigating effects of magnesium supplementation^14,20–23^. In prediabetic patients with hypomagnesemia it was shown that magnesium (382 mg for 4 months) increased HDL-cholesterol and triglyceride levels significantly^14^. Meta-analysis of nine RCTs conducted on patients with T2DM found that oral magnesium supplementation for 4–16 weeks compared with placebo increased HDL-cholesterol levels by 3.09 mmol/L (3.09 mg/dL). There was no effect on total cholesterol, LDL-cholesterol, or triglyceride levels. The median dose of magnesium supplementation in the treatment groups was 15 mmol/day (360 mg/day)^21^. Moreover, in animal models, magnesium deficient diets resulted in elevated plasma total cholesterol, LDL-cholesterol, and triacylglycerol concentrations, and decreased HDL-cholesterol^24,25^.

Magnesium seems to modulate some processes related to adipogenesis, lipolysis, and inflammation^26,27^. An animal study showed that magnesium regulated the enzymatic activity and transcriptional genes related to lipid metabolism and reduced the accumulation of lipids in hepatocytes^27^. Increasing dietary magnesium content was associated with increased expression of triacylglycerol lipase in adipose tissue, which is key in degrading triacylglycerols. Moreover, genes involved in lipogenesis, such as glucose-6-phosphate dehydrogenase, showed decreased activity in the liver^27^. Magnesium also stimulates lipoprotein lipase activity in the muscles, which lowers the concentration of triacylglycerols while increasing HDL-cholesterol levels^28^.

In this study, no significant effect was observed on fasting plasma glucose and insulin levels, HbA1c and HOMA-IR index following magnesium supplementation. Findings of the studies assessing the effects of magnesium supplements on insulin sensitivity and glucose control have been inconsistent. One study done on people with metabolic syndrome showed that taking 400 mg of magnesium daily for 12 weeks did not reduce insulin resistance or improve metabolic control^13^. In another study, taking 382 mg magnesium for 4 months resulted in reduced blood sugar levels and HOMA-IR index in individuals with prediabetes. In addition, a meta-analysis and systematic review of 21 randomized trials assessed the effects of magnesium supplements on glucose, insulin, and HOMA-IR index in both diabetics and non-diabetics. The included clinical trials used different doses of magnesium supplements (ranging from 300 to 600 mg/day) in different lengths of time (from 1 to 6 months). Meta-analysis of RCTs showed that oral magnesium supplementation significantly improved the HOMA-IR index, while it had no significant effect on levels of glucose, insulin, and HbA1c in diabetics and nondiabetics. However, in a subgroup analysis comparing magnesium supplementation durations of less than 4 months versus equal to or greater than 4 months, magnesium supplementation significantly reduced both the fasting glucose levels and the HOMA-IR index among the studies with supplementation periods equal to or exceeding 4 months versus shorter periods of supplementation^29^. A finding that indicates the importance of long-term magnesium supplementation to achieve glycemic control benefits. Finally, a recent meta-analysis investigating the effect of oral magnesium supplementation on glucose parameters in participants at high risk of diabetes inconsistent with our study showed magnesium supplementation significantly improved fasting plasma glucose and after a 2 h oral glucose tolerance test^30^. 12 randomized clinical trials were included in the meta-analysis, but only two were conducted in prediabetes, and 10 studies had participants with different conditions like obesity, metabolic syndrome, and polycystic ovary. In addition, many studies included in the meta-analysis used magnesium doses exceeding 300 mg, which could have caused the difference.

The current study found no effect of magnesium on blood pressure. A lack of effect on blood pressure is in agreement with the findings of some RCTs^20,23,31^. Prediabetic adults receiving 350 mg of magnesium per day for 8 weeks did not have a change in blood pressure^20^. Another study reported that long-term magnesium supplementation (350 mg daily for 24 weeks) did not change office and 24-h ambulatory blood pressure levels in overweight and obese adults^31^. In healthy young men with a family history of metabolic syndrome, magnesium supplements had no beneficial effect on blood pressure levels^23^. However, the results of RCTs on magnesium supplementation and blood pressure have been inconsistent and some studies found positive effects on blood pressure^32,33^.

In addition, no beneficial effects were found on CRP and uric acid levels in our study. Some RCTs examining the effects of magnesium supplementation on CRP reported similar results^13,22,23,32^. A recent meta-analysis of 18 studies concluded that magnesium supplementation did not significantly change CRP levels in serum. Moreover, analysis of subgroups showed no effects related to duration of intervention, magnesium dosage, gender, or participants' health condition on the effect of oral magnesium supplementation on CRP levels^34^.

Finally, based on our findings, weight, BMI, and triglyceride levels decreased in both study groups, while insulin, HOMA-IR, and CRP levels increased at the end of the study. Weight loss is generally associated with improvements in insulin resistance^35,36^. This relationship, however, is not supported by all evidence. A recent randomized controlled trial investigating the effects of weight loss with or without exercise on insulin sensitivity found to significantly improve insulin sensitivity, calorie restriction alone is not sufficient and exercise is required in addition. Exercise with weight loss improved insulin sensitivity, decreased ectopic fat, and preserved lean mass and strength. However, weight loss alone decreased lean mass and strength. Therefore, cardiometabolic benefits of weight loss may not occur with weight loss alone^37^. In our study, participants were not advised to do more physical activity and were not supported to change their usual physical activity habits. It might explain why insulin resistance has not improved. Additionally, there is the possibility that people with prediabetes require more weight loss to achieve cardiometabolic benefits. Also, BMI changes are usually correlated with CRP changes^38,39^, but this correlation is not constant^40^. According to a study on women with poly cystic ovary, losing 4–5% of their bodies weight was not effective in lowering CRP concentrations, suggesting that a greater weight loss, abdominal or visceral adiposity may be needed^40^.

Magnesium concentrations in intracellular fluid were not assessed in our study. In light of the fact that magnesium is mainly an intracellular ion, serum measurements may not be a useful tool for assessing magnesium status. Thus, serum magnesium was not tested and the lack of information on intracellular magnesium concentrations is a major limitation of this study. Also, we lacked information regarding magnesium intake in the diet and physical activity. However, by asking the participants to maintain their habitual diet and physical activity, this is less likely to be an issue. Our study had some strengths, including the randomized design, which provides the most solid basis for determining causality. In addition, this study investigated the effect of magnesium supplementation on a wide range of the cardiometabolic markers in people with prediabetes simultaneously.

In conclusion, this study showed that magnesium supplementation increased HDL-cholesterol levels in people with prediabetes significantly, which is considered as a significant cardiometabolic factor in the prevention of cardiovascular diseases in prediabetic patients who have a high risk of cardiovascular diseases in the future. However, other cardiometabolic markers were not improved by magnesium supplementation at the above dosage and duration. Considering possible limitations regarding magnesium formulation, duration of therapy and limited sample size, we must be cautious before drawing any definitive conclusions. Then, further studies using different doses of magnesium and longer intervention periods would be the next step to assess the effects of magnesium on cardiometabolic markers.

## Data Availability

The datasets generated and/or analysed during the current study are available from the corresponding author on reasonable request.

## References

[1] Eckel RH (2011). Obesity and type 2 diabetes: What can be unified and what needs to be individualized?. Diabetes Care.

[2] Phillips LS, Ratner RE, Buse JB, Kahn SE (2014). We can change the natural history of type 2 diabetes. Diabetes Care.

[3] American Diabetes Association Professional Practice Committee (2021). 10. Cardiovascular disease and risk management: Standards of Medical Care in Diabetes—2022. Diabetes Care.

[4] American Diabetes Association Professional Practice Committee (2021). 11. Chronic kidney disease and risk management: Standards of Medical Care in Diabetes—2022. Diabetes Care.

[5] American Diabetes Association Professional Practice Committee (2021). 12. Retinopathy, neuropathy, and foot care: Standards of Medical Care in Diabetes—2022. Diabetes Care.

[6] Seko Y (2018). Insulin resistance increases the risk of incident type 2 diabetes mellitus in patients with non-alcoholic fatty liver disease. Hepatol. Res..

[7] Liamis G, Liberopoulos E, Barkas F, Elisaf M (2014). Diabetes mellitus and electrolyte disorders. World J. Clin. Cases.

[8] Pham PC, Pham PM, Pham SV, Miller JM, Pham PT (2007). Hypomagnesemia in patients with type 2 diabetes. Clin. J. Am. Soc. Nephrol..

[9] Gröber U, Schmidt J, Kisters K (2015). Magnesium in prevention and therapy. Nutrients.

[10] Volpe SL (2013). Magnesium in disease prevention and overall health. Adv. Nutr..

[11] Günther T (2010). The biochemical function of Mg2+ in insulin secretion, insulin signal transduction and insulin resistance. Magnes. Res..

[12] Simental-Mendía LE, Rodríguez-Morán M, Guerrero-Romero F (2014). Oral magnesium supplementation decreases C-reactive protein levels in subjects with prediabetes and hypomagnesemia: A clinical randomized double-blind placebo-controlled trial. Arch. Med. Res..

[13] Lima de Souza ESML (2014). Magnesium replacement does not improve insulin resistance in patients with metabolic syndrome: A 12-week randomized double-blind study. J. Clin. Med. Res..

[14] Guerrero-Romero F, Simental-Mendía LE, Hernández-Ronquillo G, Rodriguez-Morán M (2015). Oral magnesium supplementation improves glycaemic status in subjects with prediabetes and hypomagnesaemia: A double-blind placebo-controlled randomized trial. Diabetes Metab..

[15] Chatterjee A (2012). Managing cardiometabolic risk in primary care: Summary of the 2011 consensus statement. Can. Fam. Phys..

[16] Safari S, Amini M, Aminorroaya A, Feizi A (2020). Patterns of changes in serum lipid profiles in prediabetic subjects: Results from a 16-year prospective cohort study among first-degree relatives of type 2 diabetic patients. Lipids Health Dis..

[17] American Diabetes Association Professional Practice Committee (2021). 2. Classification and diagnosis of diabetes: Standards of Medical Care in Diabetes—2022. Diabetes Care.

[18] Bickley LS, Szilagyi PG, Hoffman RM, Soriano RP (2020). Bates' Guide to Physical Examination and History Taking.

[19] Wallace T, Levy J, Matthews D (2004). Use and abuse of HOMA modelling. Diabetes Care.

[20] Ham JY, Shon YH (2020). Natural magnesium-enriched deep-sea water improves insulin resistance and the lipid profile of prediabetic adults: A randomized, double-blinded crossover trial. Nutrients..

[21] Song Y, He K, Levitan EB, Manson JE, Liu S (2006). Effects of oral magnesium supplementation on glycaemic control in type 2 diabetes: A meta-analysis of randomized double-blind controlled trials. Diabet. Med..

[22] Joris PJ, Plat J, Bakker SJ, Mensink RP (2017). Effects of long-term magnesium supplementation on endothelial function and cardiometabolic risk markers: A randomized controlled trial in overweight/obese adults. Sci. Rep..

[23] Cosaro E (2014). Effects of magnesium supplements on blood pressure, endothelial function and metabolic parameters in healthy young men with a family history of metabolic syndrome. Nutr. Metab. Cardiovasc. Dis..

[24] Luthringer C, Rayssiguier Y, Gueux E, Berthelot A (1988). Effect of moderate magnesium deficiency on serum lipids, blood pressure and cardiovascular reactivity in normotensive rats. Br. J. Nutr..

[25] Rayssiguier Y, Gueux E, Weiser D (1981). Effect of magnesium deficiency on lipid metabolism in rats fed a high carbohydrate diet. J. Nutr..

[26] Altura BM (2010). Magnesium deficiency upregulates serine palmitoyl transferase (SPT 1 and SPT 2) in cardiovascular tissues: Relationship to serum ionized Mg and cytochrome c. Am. J. Physiol. Heart Circ. Physiol..

[27] Wei CC (2017). Magnesium reduces hepatic lipid accumulation in yellow catfish (*Pelteobagrus fulvidraco*) and modulates lipogenesis and lipolysis via PPARA, JAK-STAT, and AMPK pathways in hepatocytes. J. Nutr..

[28] Dos Santos LR (2021). Cardiovascular diseases in obesity: What is the role of magnesium?. Biol. Trace Elem. Res..

[29] Simental-Mendía LE, Sahebkar A, Rodríguez-Morán M, Guerrero-Romero F (2016). A systematic review and meta-analysis of randomized controlled trials on the effects of magnesium supplementation on insulin sensitivity and glucose control. Pharmacol. Res..

[30] Veronese N (2021). Oral magnesium supplementation for treating glucose metabolism parameters in people with or at risk of diabetes: A systematic review and meta-analysis of double-blind randomized controlled trials. Nutrients..

[31] Joris PJ, Plat J, Bakker SJ, Mensink RP (2016). Long-term magnesium supplementation improves arterial stiffness in overweight and obese adults: Results of a randomized, double-blind, placebo-controlled intervention trial. Am. J. Clin. Nutr..

[32] Simental-Mendía LE, Rodríguez-Morán M, Reyes-Romero MA, Guerrero-Romero F (2012). No positive effect of oral magnesium supplementation in the decreases of inflammation in subjects with prediabetes: A pilot study. Magnes. Res..

[33] Rodríguez-Moran M, Guerrero-Romero F (2014). Oral magnesium supplementation improves the metabolic profile of metabolically obese, normal-weight individuals: A randomized double-blind placebo-controlled trial. Arch. Med. Res..

[34] Talebi S, Miraghajani M, Hosseini R, Mohammadi H (2022). The effect of oral magnesium supplementation on inflammatory biomarkers in adults: A comprehensive systematic review and dose-response meta-analysis of randomized clinical trials. Biol. Trace Elem. Res..

[35] Clamp LD, Hume DJ, Lambert EV, Kroff J (2017). Enhanced insulin sensitivity in successful, long-term weight loss maintainers compared with matched controls with no weight loss history. Nutr. Diabetes.

[36] Kopp HP (2003). Impact of weight loss on inflammatory proteins and their association with the insulin resistance syndrome in morbidly obese patients. Arterioscler. Thromb. Vasc. Biol..

[37] Brennan AM (2022). Weight loss and exercise differentially affect insulin sensitivity, body composition, cardiorespiratory fitness, and muscle strength in older adults with obesity: A randomized controlled trial. J. Gerontol. Biol. Sci. Med. Sci..

[38] Yatsuya H (2011). Changes in C-reactive protein during weight loss and the association with changes in anthropometric variables in men and women: LIFE Study. Int. J. Obes. (Lond.).

[39] Belalcazar LM (2010). A 1-year lifestyle intervention for weight loss in individuals with type 2 diabetes reduces high C-reactive protein levels and identifies metabolic predictors of change: From the look AHEAD (action for health in diabetes) study. Diabetes Care.

[40] Moran LJ (2007). C-reactive protein before and after weight loss in overweight women with and without polycystic ovary syndrome. J. Clin. Endocrinol. Metab..

